# Integrated proteomic and metabolomic profiling of lymph after trauma-induced hypercoagulopathy and antithrombotic therapy

**DOI:** 10.1186/s12959-024-00634-3

**Published:** 2024-07-10

**Authors:** Yangkang Zheng, Pengyu Wang, Lin Cong, Qi Shi, Yongjian Zhao, YongJun Wang

**Affiliations:** 1 Longhua Hospital, Shanghai University of Traditional Chinese Medicine, 725 Wan-Ping South Road, Shanghai, 200032 China; 2 Spine Institute, Shanghai University of Traditional Chinese Medicine, 725 Wan-Ping South Road, Shanghai, 200032 China; 3 Key Laboratory of Theory and Therapy of Muscles and Bones, Ministry of Education, Shanghai University of Traditional Chinese Medicine), 1200 Cailun Road, Shanghai, 201203 China; 4 Department of Biochemistry and Molecular Cell Biology, School of Medicine, Shanghai Jiao Tong University, Shanghai, 200025 China

**Keywords:** Trauma-induced hypercoagulopathy, Thoracic duct lymph, Proteomics, Untargeted metabolomics, Antithrombotic therapy

## Abstract

**Background:**

Routine coagulation tests are not widely accepted diagnostic criteria of trauma-induced hypercoagulopathy (TIH) due to insensitivity. Lymphatic vessels drain approximately 10% of the interstitial fluid into the lymphatic system and form lymph.

**Subjective:**

The purpose of this study was to identify the potential lymph biomarkers for TIH.

**Methods:**

Eighteen male Sprague-Dawley rats were randomly assigned to the sham (non-fractured rats with sham surgery and vehicle treatment), the VEH (fractured rats with vehicle treatment) and the CLO (fractured rats with clopidogrel treatment) group. Thoracic duct lymph was obtained to perform proteomics and untargeted metabolomics.

**Results:**

A total of 1207 proteins and 16,695 metabolites were identified. The top 5 GO terms of lymph proteomics indicated that oxidative stress and innate immunity were closely associated with TIH and antithrombotic therapy. The top 5 GO terms of lymph metabolomics showed that homocystine and lysophosphatidylcholine were the differential expressed metabolites (DEMs) between the sham and VEH groups, while cholic acid, docosahexaenoic acid, N1-Methyl-2-pyridone-5-carboxamide, isoleucine and testosterone are the DEMs between the VEH and CLO group.

**Conclusions:**

This study presents the first proteomic and metabolomic profiling of lymph after TIH and antithrombotic therapy, and predicts the possible lymph biomarkers for TIH.

**Supplementary Information:**

The online version contains supplementary material available at 10.1186/s12959-024-00634-3.

## Introduction

Trauma-induced coagulopathy (TIC) is a dynamic and complex coagulation dysfunction, characterized by hypocoagulability in the early hours, resulting in hemorrhagic shock, and hypercoagulability following the hypocoagulable state, resulting in venous thromboembolism and multiple organ failure [1]. As a result, TIC is regarded as an independent risk factor for poor prognosis among trauma patients [2–5]. Most studies on TIC mainly focus on hypocoagulability, however, 22.2–85.1% of trauma patients within days of injury develop trauma-induced hypercoagulopathy (TIH). TIH raises the risk of thrombotic events and mortality by 2–4 times [3, 6, 7]. Hence, discovering the potential biomarkers of TIH is essential for medical practice [4].

Routine coagulation tests, such as prothrombin time (PT), activated partial prothrombin time (APTT), D-dimers (DD) and fibrinogen (or fibrin) degradation products (FDPs), are suggested as the diagnostic indicators of TIH. However, these are not widely accepted diagnostic criteria of TIH due to the insensitivity [4]. Lymphatic vessels, as the second circulatory pathway, drain approximately 10% of the interstitial fluid into the lymphatic system and form lymph [8]. At the end of lymphatic draining pathways, most lymph is returned to the circulatory system through the thoracic duct. In contrast to blood vessels, lymphatic vessels play a vital role in macromolecule transport, lipid metabolism and waste clearance [9–13]. Previous research also demonstrates that lymph is not a plasma ultrafiltrate and presents some unique substances [14]. Therefore, to compensate for the shortcomings of serum and plasma, lymph might be a potential and valuable source of novel biomarkers for TIH.

Our previous investigation found that lymphatic drainage insufficiency was caused by a significant amount of lymphatic platelet thrombosis (LPT) blocking lymphatic vessels on the first day post -traumatic fracture [15]. By removing LPT, a low dose of clopidogrel rapidly restored lymphatic drainage function and alleviated early fracture complications [15]. These results suggest that traumatic fracture on the first day causes TIH, which is not only manifested with deep venous thrombosis, but also with LPT. Therefore, to explore the early constitutive alterations in lymph after TIH, we utilized integrated proteomic and metabolomic analysis of lymph in the sham group (non-fractured rats with sham surgery and vehicle treatment) and the vehicle (VEH) group (fractured rats with vehicle treatment). Furthermore, to find possible TIH biomarkers and unveil the lymphatic clearance mechanism treated with a blood thinner, we employed integrated proteomic and metabolomic analysis of lymph in the VEH group and clopidogrel (CLO) group (fractured rats with clopidogrel treatment).

## Methods

### Animals

This study was approved by Shanghai University of Traditional Chinese Medicine-Animal Ethics Committee (PZSHUTCM211101023). Eighteen 8 ~ 10-week-old Sprague-Dawley rats ( Shanghai Jessie Experimental Animal Co., Ltd ) were kept in isolation cages in the animal center with a 12-hour light/dark cycle. All rats were fed with regular rodent’s chow and sterilized tap water ad libitum. Eighteen rats were randomly assigned to three groups (*N* = 6/group) and accommodated for 1 week before experimental procedures.

### Thoracic duct lymph collection

Cannulation of the thoracic lymph duct was carried out as the previous study described [16]. Isoflurane inhalation (5% induction at a flow rate of 3 L/min, 2% maintenance at a flow rate of 1 L/min) was used to induce and sustain general anesthesia. First, to expose the thoracic duct, we made a midline abdominal incision approximately two-thirds of the length of the abdomen posterior to the xiphoid cartilage. Second, we isolated the thoracic duct from the abdominal aorta, ligated the proximal ends of the thoracic duct with two 4 − 0 silks, and cannulated the thoracic duct with a 15 cm length of polyethylene tubing primed with heparinized saline. A white lymph was seen flowing in the tubing. Third, to collect the thoracic duct lymph, we firmly fixed the polyethylene tubing at the distant ends of the thoracic duct with two 4 − 0 silk, passed the polyethylene tubing through the peritoneum and skin, and connected the polyethylene tubing to the anticoagulant tube. Following repeated peritoneal lavages, the peritoneum and skin were sutured closed. Topical anesthetic cream was applied to the rats’ suture line. Anticoagulant vacutainers need to be replaced every 2 h. At last, collected lymph samples at 24 h post-surgery were centrifuged at 1000 g for 10 min at 4 ℃ to remove insoluble cell lysates and precipitation before being stored at -20℃.

### Establishment of rat TIH model

Fracture is acknowledged as a high risk factor of TIH [17–19]. In this study, traumatic fractured rats were used to generate a TIH model. After successfully cannulating the rats’ thoracic lymph ducts, eighteen animals were randomly assigned to the sham, VEH, and CLO groups (*N* = 6/group). For the traumatic fracture-induced TIH model, the intact tibia was placed at a midpoint of two supports (30 mm apart for rat experiments). A weight of 500 g was dropped from a height of 20 cm by a modified three-point bending device. For the sham model, 6 cannulated rats were fed routinely with no operations. To simulate a real traumatic condition before hospitalization, painkillers and antibiotics were not administered to fractured rats on day 1 post-surgery.

### Antithrombolic therapy

Clopidogrel (Cat. No. HY-15,283, MedChemExpress) was dissolved in a solvent (2.5mL dimethyl sulfoxide, 15mL polyethylene glycol, 2.5mL Tween-80, and 30mL double distilled water), and intramuscularly injected near the right popliteal lymph node at 0.1 mg/kg of body weight for 5 days (once per day). For the CLO group, clopidogrel was given at 4 h after tibial fracture under the ultrasound guide. Rats in the sham and VEH groups were given the same volume of solvent.

### Sample detection for proteomics

Sample preparation procedures include abundant protein depletion by ProteoExtract^®^ Albumin Removal Kit ( Cat. No.122,640, Millipore) [20], protein digestion, and sample desalting according to Shanghai Luming Biological Technology Co., LTD’s instructions.

### Liquid chromatography-mass spectrometry

The Proteomic data analysis was performed by Shanghai OE Biotech Co., Ltd. (Shanghai, China). All analyses were performed by a Tims TOF Pro mass spectrometer (Thermo, Bruker) equipped with an EASY-nLC 1200 system (Thermo, USA). Samples were loaded by a C18 column (15 cm × 75 μm) on an EASY-nLCTM 1200 system (Thermo, USA). The flow rate was 300 nL/min and linear gradient was set as follows: 0 ~ 66 min, 3–27% B; 66 ~ 73 min, 27–46% B; 73 ~ 84 min, 46–100% B;84–90 min, 100% B. Ion mobility is set from 0.6 to 1.6 Vs/cm2 and the collision energy range from 20 to 59 eV. The MS/MS spectra were recorded from 100 to 1700 m/z.

### Database search

MS/MS spectra were searched using MaxQuant 1.6.17.0 against the Rattus norvegicus-10,116 database. Search database specific parameters are set as follows: Fixed modifications: Carbamidomethyl (C); Variable modification: Oxidation (M) and Acetyl(Protein N-term); digestion: trypsin; Precursor Qvalue cutoff: 0.01; Protein Qvalue cutoff: 0.01; Missed cleavage: 2; Quantity MS-Level: MS2.

### Statistical analyses

A total of 1207 proteins expressed were identified as belonging to the proteome of lymph in this study. The thresholds of fold change (> 1.2 or < 0.83) and *P*-value < 0.05 were used to identify differentially expressed proteins (DEPs). Then we found 41 upregulated and 21 downregulated proteins in the VEH group compared with the sham group. In addition, we found 69 upregulated and 54 downregulated proteins in the CLO group compared with the VEH group. Annotation of all identified proteins was performed using GO (http://www.blast2go.com/b2ghome; http://geneontology.org/) and KEGG pathway (http://www.genome.jp/kegg/). DEPs were further used for GO and KEGG enrichment analysis. Protein-protein interaction analysis was performed using the String (https://string-db.org/). The mass spectrometry proteomics data have been deposited to the ProteomeXchange Consortium (http://proteomecentral.proteomexchange.org) via the iProX partner repository [21, 22] with the dataset identifier PXD047692.

### Sample preparation for metabolomics

Lymph samples stored at -80 ℃ were thawed at room temperature. 80 µL of the sample was added to a 1.5 mL Eppendorf tube with 240 µL methanol/acetonitrile solution (2:1, vol/vol), containing L-2-chlorophenylalanine (2 ug/mL) dissolved in methanol as internal standard, and the tube was vortexed for 1 min. The whole samples were extracted by ultrasonic for 10 min in an ice-water bath, and stored at -40 ℃ for 30 min. The extract was centrifuged at 4 °C (13,000 rpm) for 10 min. 200 µL of supernatant in a glass vial was dried in a freeze-concentration centrifugal dryer. 300 µL mixture of methanol and water (1:4, vol/vol) were added to each sample, samples vortexed for 30 s, extracted by ultrasonic for 3 min in an ice-water bath, then placed at -40 °C for 2 h. Samples were centrifuged at 4 °C (13,000 rpm) for 10 min. The supernatants (150 µL) from each tube were collected using crystal syringes, filtered through 0.22 μm microfilters, and transferred to LC vials. The vials were stored at -80 °C until LC-MS analysis. QC samples were prepared by mixing aliquots of the all samples to form a pooled sample.

### Metabolomic LC-MS/MS analysis

Metabolomic LC–MS/MS analysis was performed using ACQUITY UPLC I-Class system (Waters Corporation, Milford, USA) coupled with Q-Exactive plus quadrupole-Orbitrap mass spectrometer (Thermo Fisher Scientific, Waltham, MA, USA). An ACQUITY UPLC HSS T3 column (1.8 μm, 2.1 × 100 mm) was employed in both positive and negative modes. The mass range was from m/z 100 to 1,200. The resolution was set at 70,000 for the full MS scans and 17,500 for HCD MS/MS scans. The Collision energy was set at 10, 20 and 40 eV. The mass spectrometer operated as follows: spray voltage, 3,800 V (+) and 3,200 V (−); sheath gas flow rate, 40 arbitrary units; auxiliary gas flow rate, 8 arbitrary units; capillary temperature, 320 °C; Probe Heater Temperature, 350 °C; S-lens RF level, 50. To provide a set of data with high repeatability, the QCs were injected at regular intervals throughout the experiments.

### Metabolomic data processing and statistical analysis

The raw LC-MS data were processed by the software Progenesis QI V3.0 (Nonlinear, Dynamics, Newcastle, UK) for baseline filtering, peak identification, integral, retention time correction, peak alignment, and normalization. The extracted data were then further processed by removing any peaks with a missing value (ion intensity = 0) in more than 50% in groups, by replacing the zero value with half of the minimum value, and by screening according to the qualitative results of the compound. Compounds with resulting scores below 36 (out of 60) points were also deemed to be inaccurate and removed. A data matrix was combined from the positive and negative ion data.

The matrix was imported in R to carry out Principle Component Analysis (PCA) to observe the overall distribution among the samples and the stability of the whole analysis process. Orthogonal Partial Least-Squares-Discriminant Analysis (OPLS-DA) and Partial Least-Squares-Discriminant Analysis (PLS-DA) were utilized to distinguish the metabolites that differ between groups. To prevent overfitting, 7-fold cross-validation and 200 Response Permutation Testing (RPT) were used to evaluate the quality of the model.

Variable Importance of Projection (VIP) values obtained from the OPLS-DA model were used to rank the overall contribution of each variable to group discrimination. A two-tailed Student’s T-test was further used to verify whether the metabolites of difference between groups were significant. Differential metabolites were selected with VIP values greater than 1.0 and *p*-values less than 0.05. Differential metabolites were further used for KEGG pathway (http://www.genome.jp/kegg/) enrichment analysis. The metabolomic data have been deposited to the MetaboLights (https://www.ebi.ac.uk/metabolights/) with the dataset identifier MTBLS9145.

### Multi-omics data integration of lymph

Pearson’s correlation tests were used to detect the associations between the differentially expressed proteins (DEPs) and differentially expressed metabolites (DEMs). The top 20 of DEPs and DEMs are selected based on VIP ranking. Common enrichment pathways of DEPs and DEMs are further presented with a Bubble plot.

### Data availability

The data that support the findings of this study are available from the authors upon reasonable request.

## Results

### Scheme of the methodology

To explore early constitutive changes in lymph and reveal the potential pharmacodynamic mechanism of antithrombotic therapy for tissue repair after TIH, a total of 18 Sprague Dawley rats were randomly assigned to three groups. The sham group has 6 non-fractured rats with sham surgery and vehicle treatment, the vehicle (VEH) group has 6 traumatic fractured rats with vehicle treatment, and the clopidogrel (CLO) group has 6 fractured rats with clopidogrel treatment. The thoracic duct lymph at 24 h post-surgery were collected for integrated proteomics by 4D-label free and untargeted metabolomics by liquid-chromatography coupled with tandem mass spectrometry (LC-MS/MS). This design was illustrated in Supplementary Fig. 1.

### Overview of proteomic data of lymph

Lymph proteomics identified 9970 peptides and 1207 proteins in total, wherein 120 proteins are more than 100 kDa in molecular weight, namely macro-molecules (Supplementary Fig. 2A). Furthermore, a total of 678 credible proteins were identified based on bioinformatics analysis. To ensure the reliability of 4D-label free, we projected their proteomic profiles onto one dot plot by principal component analysis (PCA), and one hierarchical clustering dendrogram of sample Euclidean distance (Supplementary Fig. 2B-C). 18 samples could be well clustered to respectively correspond to the sham, VEH, and CLO groups (Supplementary Fig. 2C). Samples in CLO group were closely clustered and clearly distinguished from the VEH group (Supplementary Fig. 2B). These data present good repeatability within groups and significant differences among groups. 266 proteins out of 678 credible proteins showed significant differences. DEPs were analyzed based on log2 fold change (FC) > 1.2 and adjusted *P* value < 0.05 by t-test. Compared to the sham group, 41 proteins were significantly increased while 21 proteins were significantly decreased in the VEH group (Supplementary Fig. 1D). It implies that lymphatic vessels transport traumatic fracture-induced proteins at the injured sites. Compared to the sham or VEH group, the number of significantly increased proteins was greater than the number of significantly decreased proteins in the CLO group (Supplementary Fig. 1D). This suggests that thrombolytic therapy improves lymphatic drainage function and transports more proteins expressed at fracture sites. Venn plot further analyzed the number of overlapped and unique differential proteins between two groups (Supplementary Fig. 2D). Venn plot showed that 62 (41 up-regulated and 21 down-regulated), 123 (69 up-regulated and 54 down-regulated), and 191 (137 up-regulated and 54 down-regulated) proteins were significantly changed in the lymph among the VEH group vs. the sham group, the CLO group vs. the VEH group and the CLO group vs. the sham group, respectively (Supplementary Fig. 2D).

### Proteomic analysis of lymph between the sham and VEH groups

**Fig. 1:**
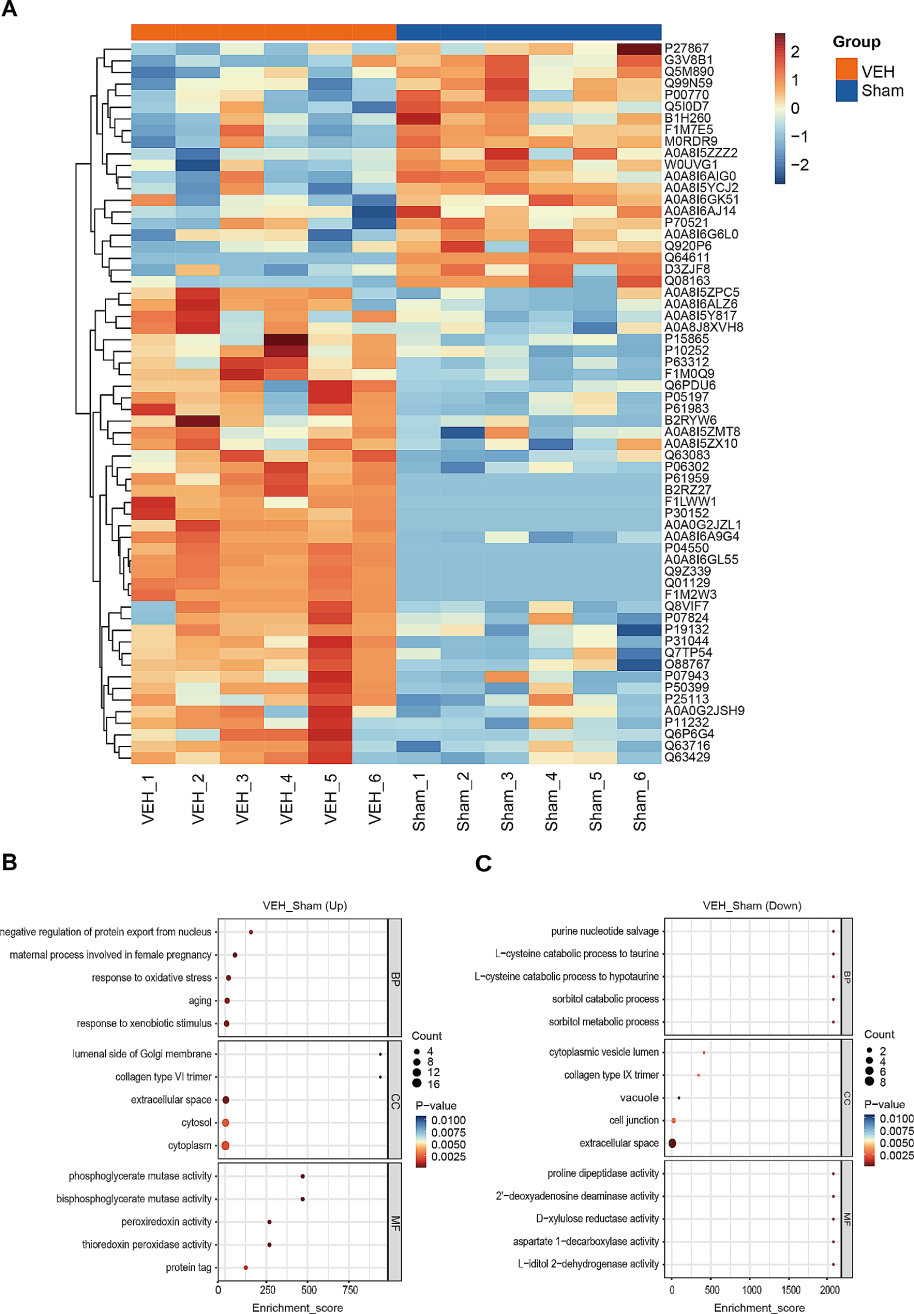
Proteomic analysis of lymph between the sham and VEH groups. **(A)** Hierarchical clustering heatmap of identified DEPs between the VEH group vs. the Sham group. Columns represent groups and rows represent proteins. Blue to red colors represent the expression level of protein from low to high. **(B)** Top 5 GO terms of biological process (BP), cellular component (CC), and molecular function (MF) enriched by the up-regulated proteins. **(C)** Top 5 GO terms of BP, CC, and MF enriched by the down-regulated proteins

62 DEPs were identified between the sham and VEH groups and presented in the hierarchical clustering heatmap (Fig. 1A). More details of these DEPs were shown in Supplemental Table 1. We further applied GO enrichment analysis of DEPs. The enriched GO terms were included by ListHits > 1 and ranked by their corresponding -log10 *P* value. The top 5 GO terms of biological process (BP) enriched by up-regulated DEPs were maternal process involved in female pregnancy, response to oxidative stress, response to xenobiotic stimulus, aging, and negative regulation of protein export from nucleus. (Fig. 1B). While the top 5 GO terms of BP enriched by down-regulated DEPs were sorbitol metabolic process, sorbitol catabolic process, L-cysteine catabolic process to hypotaurine, L-cysteine catabolic process to taurine, and purine nucleotide salvage (Fig. 1C). These results imply that TIH mainly induces multiple harmful proteins expression and oxidative stress.

### Proteomic analysis of lymph between the VEH and CLO groups

**Fig. 2:**
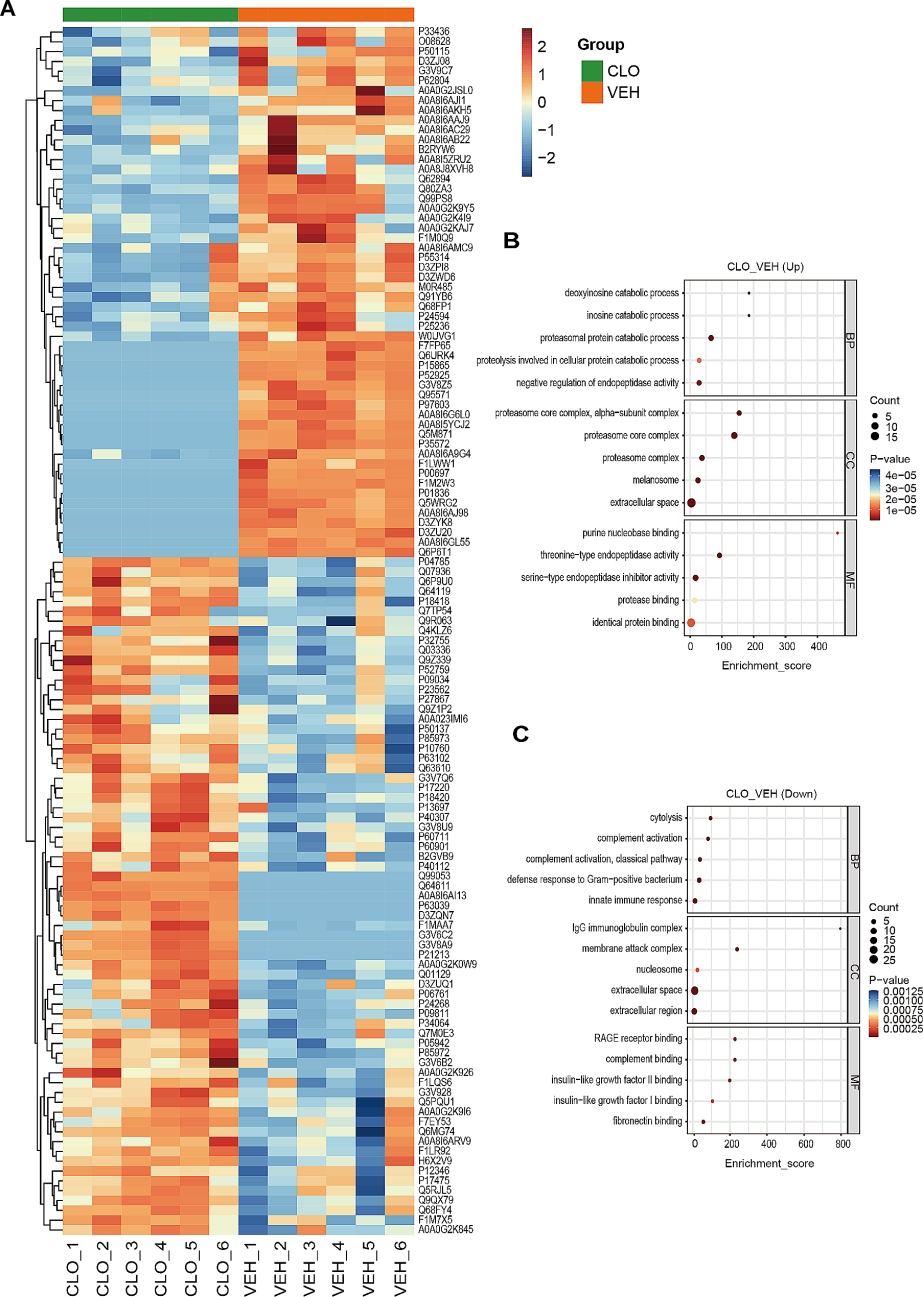
Proteomic analysis of lymph between the VEH and CLO groups. **(A)** Hierarchical clustering heatmap of identified DEPs between the CLO group vs. the VEH group. Columns represent groups and rows represent proteins. Blue to red colors represent the expression level of protein from low to high. **(B)** Top 5 GO terms of BP, CC, and MF enriched by the up-regulated proteins. **(C)** Top 5 GO terms of BP, CC, and MF enriched by the down-regulated proteins

Our previous study found that a large amount of aggregated platelets forms thrombosis within lymphatic vessels and impeded lymphatic drainage function [15]. A low dose of clopidogrel resolved lymphatic platelet thrombosis (LPT) and alleviated the early complications of bone fracture via lymphatic transporting damage-associated molecular patterns (DAMPs) [15]. To reveal the pharmacodynamic mechanism and identify the potential protein markers of antithrombotic therapy for TIH, we identified 123 DEPs between VEH and CLO groups by hierarchical clustering heatmap (Fig. 2A). More details of DEPs were shown in Supplemental Table 1. We further applied GO enrichment analysis of DEPs. The enriched GO terms were included by ListHits > 1 and ranked by their corresponding -log10 *P* value. The top 5 GO terms of BP enriched by up-regulated DEPs were proteasomal protein catabolic process, negative regulation of endopeptidase activity, proteolysis involved in cellular protein catabolic process, inosine catabolic process, and deoxyinosine catabolic process (Fig. 2B). While the top 5 GO terms of BP enriched by down-regulated DEPs were defense response to Gram-positive bacterium, innate immune response, complement activation, classical pathway, cytolysis, and complement activation (Fig. 2C). These results suggest antithrombotic therapy systematically alleviate excessive inflammatory and immune responses after TIH.

### Overview of metabolomic data of lymph

A total of 16,695 metabolites were identified and they were primarily composed of carboxylic acids, fatty acyls, and derivatives (Supplementary Fig. 3B). To ensure the reliability of untargeted metabolomic data of lymph, we analyzed metabolomic data of lymph with metabolites intensity distribution of quality control (QC) principal component analysis (PCA), orthogonal partial least squares analysis (OPLS-DA), and permutation analysis. The metabolites intensity distribution of quality QC samples showed the median line of the QC sample was approximately at the same level, indicating good stability and reproducibility of this experiment (Supplementary Fig. 3A). PCA plot showed that QC samples were closely clustered together, demonstrating the good stability of LC-MS (Supplementary Fig. 3C). Given the significant differences within the group, we further utilized OPLS-DA and permutation analysis to compare the difference between the sham &VEH groups, and the VEH&CLO groups. The results indicate a significant separation between the VEH group and sham group, and likewise between the VEH group and CLO group (Supplementary Fig. 3D-E). In addition, the results of permutation tests showed no over-fitting of the LC-MS data and suggest that the OPLS-DA model is valid (Supplementary Fig. 3E). To sum up, the lymph of the VEH group is different from the sham and CLO groups, respectively. The DEMs were analyzed based on *P-*value < 0.05 by t-test and of variable important in projection (VIP) > 1. 80 DEMs (46 up-regulated and 34 down-regulated), 116 DEMs (37 up-regulated and 79 down-regulated), and 210 DEMs (75 up-regulated and 135 down-regulated) were identified in the lymph among the VEH group vs. the sham group, the CLO vs. the VEH group, and the CLO group vs. the sham group, respectively (Supplementary Fig. 3F). Venn plot analyzed the number of overlapped and unique DEMs between two groups (Supplementary Fig. 3G).

### Metabolomic analysis of lymph between the sham and VEH groups

**Fig. 3:**
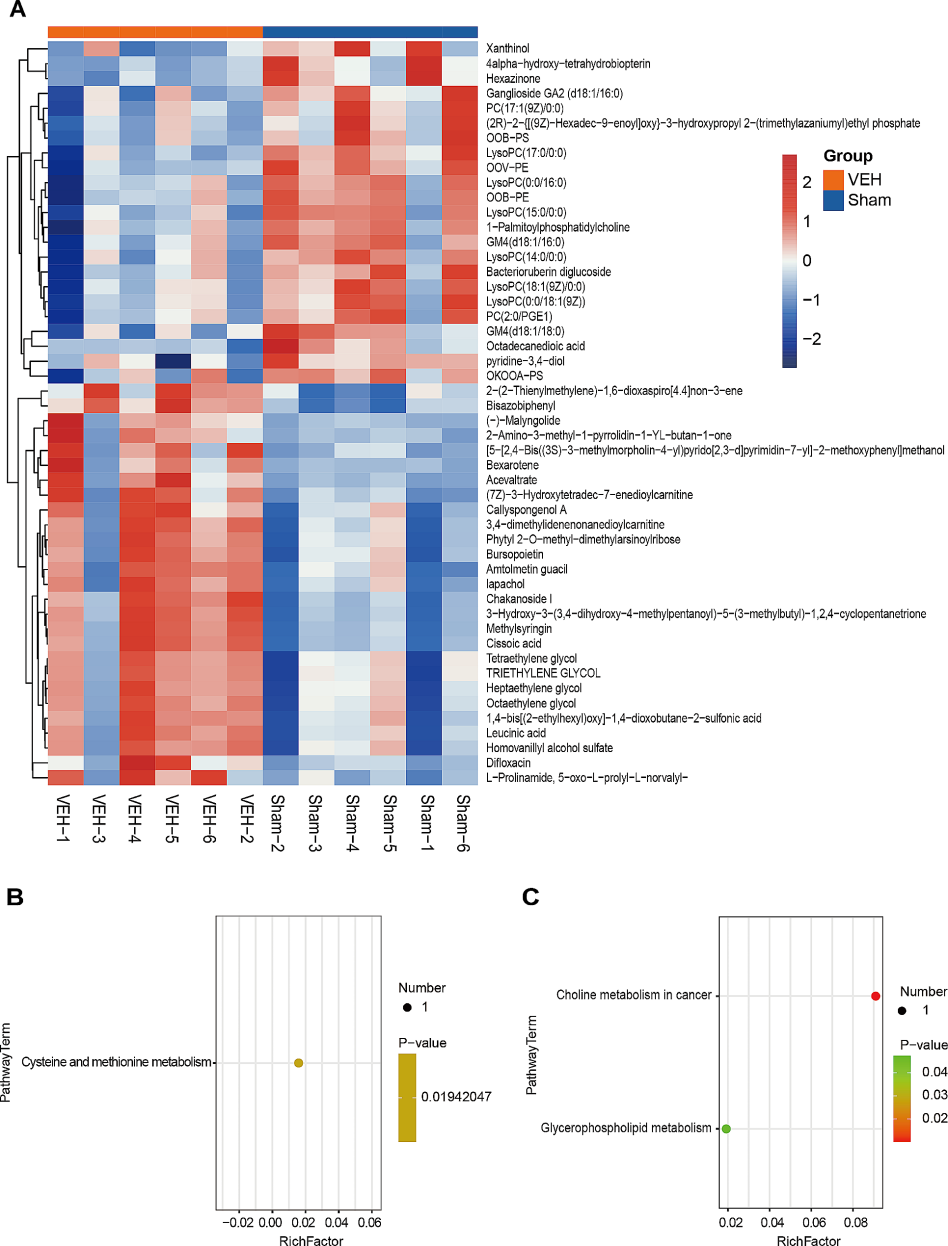
Metabolomic analysis of lymph between the sham and VEH groups. **(A)** Hierarchical clustering heatmap of identified metabolites. Columns represent groups and rows represent proteins. Blue to red colors represent the expression level of protein from low to high. **(B)** Bubble plot of the significant enrichment pathways of up-regulated metabolites. **(C)** Bubble plot of the significant enrichment pathways of down-regulated metabolites. Rich Factor is defined as the number of differential metabolites annotated to the pathways divided by all identified metabolites annotated to the pathway

We identified 80 DEMs between the sham and VEH groups. The hierarchical clustering heatmap further revealed the top 50 DEMs by VIP ranking (Fig. 3A). More details of DEMs were shown in Supplemental Table 2. We further conducted a KEGG pathway enrichment analysis of DEMs with a *P*-value less than 0.05. The pathway term enriched by up-regulated DEM was cysteine and methionine metabolism (Fig. 3B). The DEM was L-Homocystine. While the pathway terms enriched by down-regulated DEMs were choline metabolism in cancer and glycerophospholipid metabolism (Fig. 3C). These two pathways share the same DEMs, namely LysoPC (14:0/0:0), LysoPC (15:0/0:0), and LysoPC (17:0/0:0). This data depicts the metabolic profiling of lymph of rats with TIH.

### Metabolomic analysis of lymph between the VEH and CLO groups

**Fig. 4:**
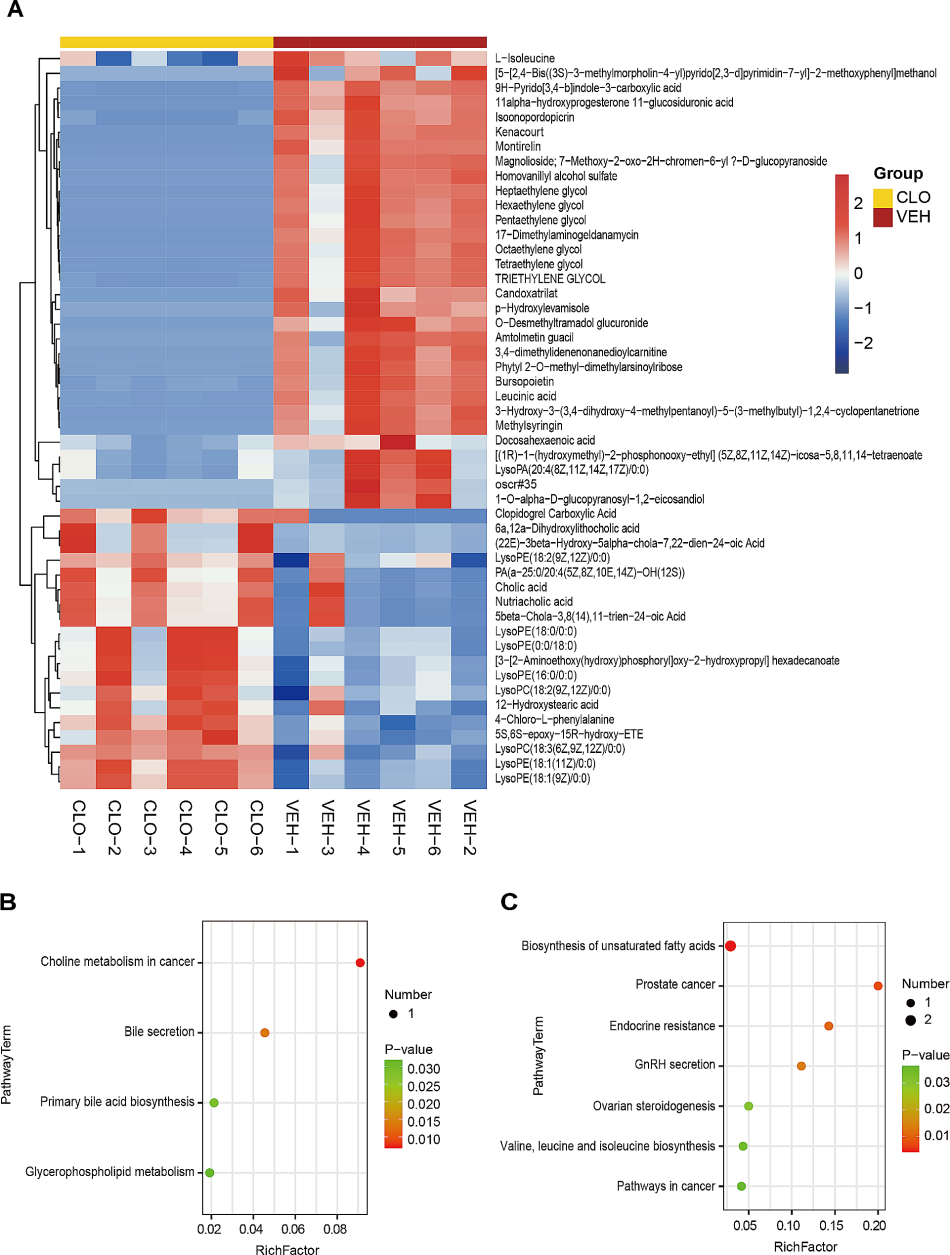
Metabolomic analysis of lymph between the VEH and CLO groups. **(A)** Hierarchical clustering heatmap of identified metabolites. Columns represent groups and rows represent proteins. Blue to red colors represent the expression level of protein from low to high. **(B)** Bubble plot of the significant enrichment pathways of up-regulated metabolites. **(C)** Bubble plot of the significant enrichment pathways of down-regulated metabolites. Rich Factor is defined as the number of differential metabolites annotated to the pathways divided by all identified metabolites annotated to the pathway

We identified 116 DEMs between the VEH and CLO groups. More details of DEMs were shown in Supplemental Table 2. We further conducted KEGG pathway enrichment analysis of DEMs with *P*-value less than 0.05. The hierarchical clustering heatmap revealed the top 50 DEMs by VIP ranking (Fig. 4A). The pathway terms enriched by up-regulated DEMs were bile secretion, primary bile acid biosynthesis, choline metabolism in cancer, and glycerophospholipid metabolism (Fig. 4B). The DEM of bile secretion and primary bile acid biosynthesis was cholic acid. The DEM of choline metabolism in cancer and glycerophospholipid metabolism was LysoPC (18:3/0:0). While the pathway terms enriched by down-regulated DEMs were biosynthesis of unsaturated fatty acids, prostate cancer, endocrine resistance, GnRH secretion, ovarian steroidogenesis, valine, leucine and isoleucine biosynthesis, pathways in cancer, valine, leucine and isoleucine degradation, aminoacyl-tRNA biosynthesis, nicotinate and nicotinamide metabolism and steroid hormone biosynthesis (Fig. 4C). Their DEMs were L-isoleucine, docosahexaenoic acid, dihomo-alpha-linolenic acid, testosterone, and N1-Methyl-2-pyridone-5-carboxamide. These results provide the potential pharmacodynamic mechanism and metabolic markers of antithrombotic therapy for TIH.

### Integrated proteomic and metabolomic analysis of lymph

**Fig. 5:**
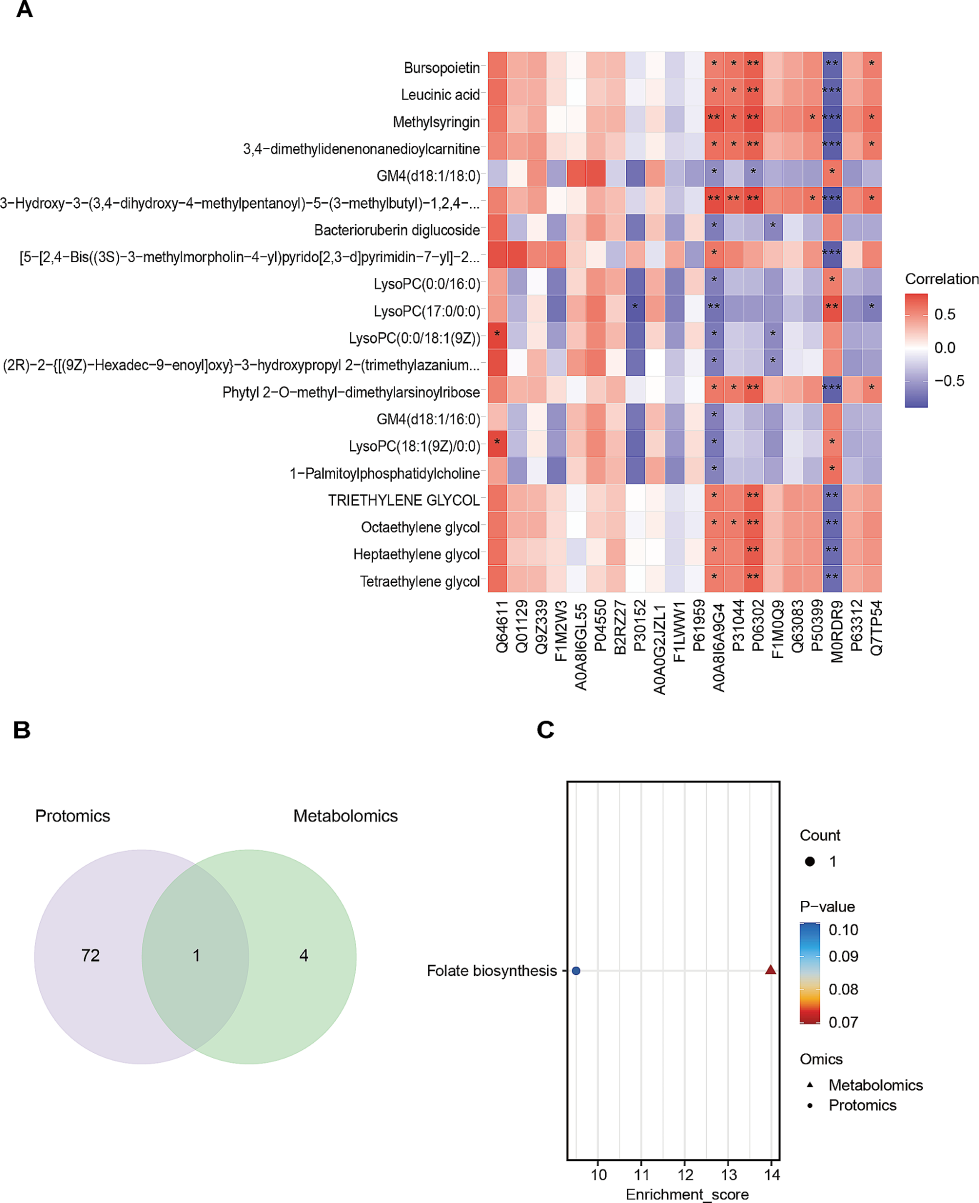
Integrated analysis of lymph between the sham and VEH groups. **(A)** Pearson’s correlation analysis of the top 20 differential proteins and metabolites based on VIP ranking between the sham and VEH groups. **(B)** Venn plot on the number of co-pathways between the sham and VEH groups enriched by the total DEPs and DEMs. **(C)** Bubble plot of the co-pathways between the VEH group and the sham group enriched by the total DEPs and DEMs. *P < 0.05, **P < 0.01 and ***P < 0.001

**Fig. 6:**
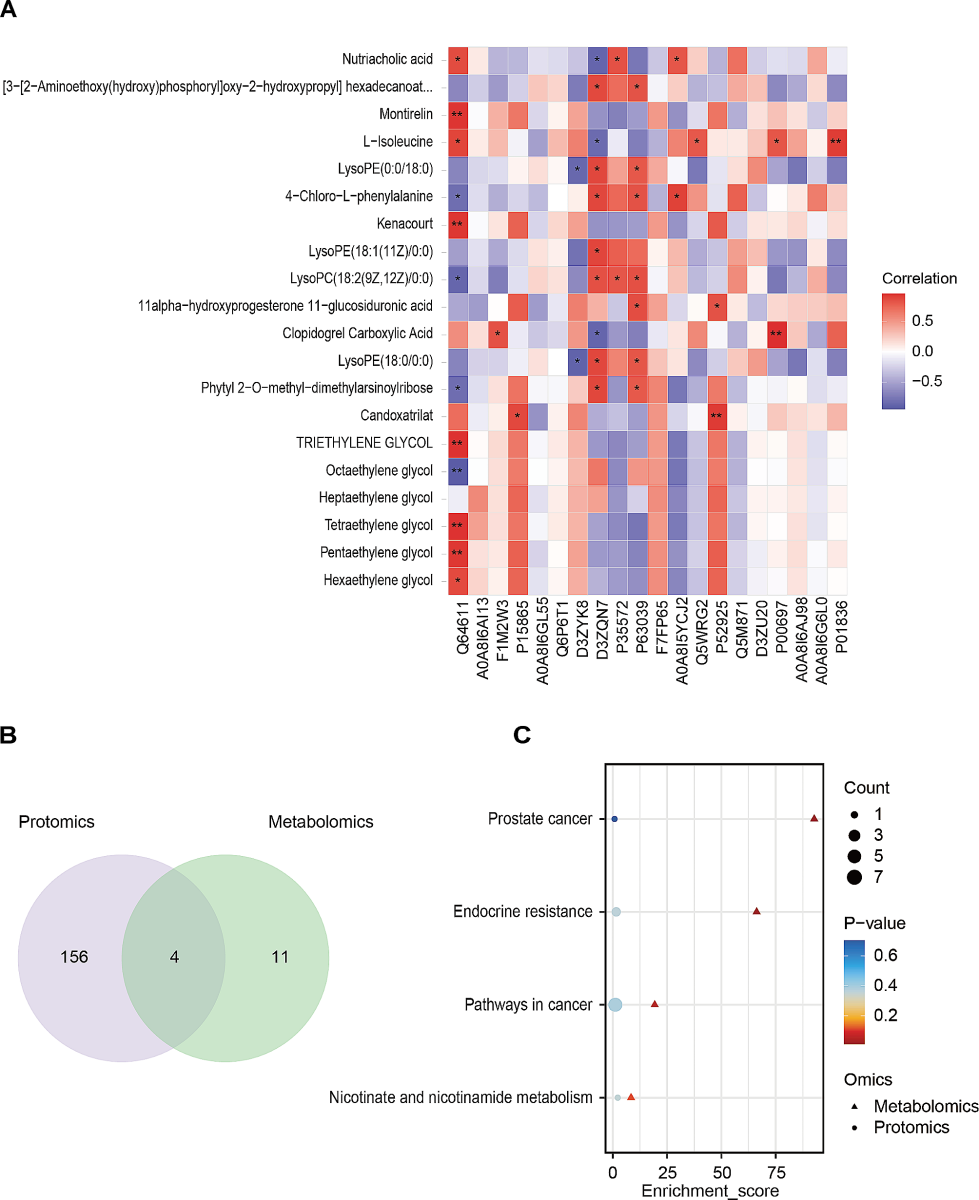
Integrated analysis of lymph between the VEH and CLO groups. **(A)** Pearson’s correlation analysis of the top 20 differential proteins and metabolites based on VIP ranking between the VEH and CLO groups. **(B)** Venn plot on the number of co-pathways between the VEH and CLO groups enriched by the total DEPs and DEMs. **(C)** Bubble plot of the co-pathways between the VEH and CLO groups enriched by the total DEPs and DEMs. *P < 0.05, **P < 0.01 and ***P < 0.001

Proteins and metabolites in life have close interaction and mutual regulatory relationship. To provide more comprehensive biological information, we constructed an integrated proteomic and metabolomic analysis of lymph for TIH. Based on the VIP ranking of DEPs and DEMs, we included the top 20 DEPs and DEMs to analyze the correlation between the sham and VEH groups by Pearson correlation algorithm (Fig. 5A). Then we respectively perform KEGG pathway enrichment of the total DEPs and DEMs between the sham and VEH groups. Venn plot indicated that 73 KEGG pathways were enriched by the total DEPs and 5 KEGG pathways were enriched by total DEMs (Fig. 5B). Furthermore, bubble plot showed that the common enrichment pathway of DEPs and DEMs between the VEH group and sham group is folate biosynthesis (Fig. 5C). Likewise, Pearson’s correlation analysis of the top 20 DEPs and DEMs between the VEH and CLO groups were shown (Fig. 6A). Venn plot indicated that 160 KEGG pathways were enriched by the total DEPs and 15 KEGG pathways were enriched by the total DEMs (Fig. 6B). Furthermore, the bubble plot showed that the common enrichment pathway of DEPs and DEMs between the VEH group and CLO group are prostate cancer, endocrine resistance, pathways in cancer, and nicotinate and nicotinamide metabolism (Fig. 6C).

## Discussion

Multi-omic analysis of lymph for TIH is rare, perhaps due to the following two reasons. (1) Thoracic duct cannulation is more complex and invasive than blood sampling [23]. Anesthesiologists and critical care specialists frequently use this medical technique to treat severely ill patients. (2) TIH received little attention from researchers and physicians since they were primarily concerned with the early stages of TIC, such as trauma-induced hypocoagulability and hemorrhagic shock [5]. Our previous research shows that a low dose of clopidogrel reduces LPT and promotes bone repair via unblocking lymphatic transport of damage-associated molecular patterns (DAMPs); additionally, unblocked lymphatic vessels exclusively transport high-weight molecular DAMPs compared to the venous system [15]. This suggests that lymph is a promising and appealing source of biofluids for disease prediction, diagnosis, and treatment. We added a larger sample size of rats with TIH and collected their lymph to be sent for multi-omic analysis. We offer the first lymph profiling of protein and metabolite alterations, predict potential lymph biomarkers for TIH and antithrombotic therapy, and explain the possible pharmacodynamic mechanism of antithrombotic therapy for tissue healing.

### Insight of lymph proteome

The lymph proteome highlights oxidative stress and innate immunity play important roles in TIH. Given the presence of overlapping DEPs in the top 5 Go terms of BP, we will focus on the link between the key DEPs and TIH in the discussion.

Monika et al. analyzed mesenteric lymph and plasma from traumatized or severely ill individuals using LC/MC [14]. Approximately 155 proteins were identified as uniquely existing in the lymph, including extracellular matrix-related proteins, actin cytoskeleton reorganization markers, and pancreatic proteins [14]. In our study, O88767 (Parkinson disease protein 7 homolog, Park7), P05197 (Elongation factor 2, Eef2), P07824 (Arginase-1, Arg1), P11232 (Thioredoxin, Txn), P30152 (Neutrophil gelatinase-associated lipocalin, Lcn2), P31044 (Phosphatidylethanolamine-binding protein 1, Pebp1), Q01129 (Decorin, Dcn), Q63716 (Peroxiredoxin-1, Prdx1), P07943 (Aldo-keto reductase family 1 member B1, Akr1b1) are the up-regulated proteins enriched in top 5 GO terms of BP (Fig. 1B). Monika’s proteomic data [14] suggests that wounded patients’ lymph, rather than plasma, may include Park7, Pebp1, and Prdx1. PARK7 is abundantly expressed in the brain, skeletal muscle, and adrenal gland (BioProject: PRJNA280600) and regulates mitochondrial dysfunction and oxidative stress [24, 25]. PEBP1, a tiny scaffold protein, inhibits protein kinase cascades and promotes ferroptosis cell death by binding with 15-lipoxygenases (15-LO) to produce hydroperoxy-PE [26]. Prdx1 is an enzyme with several functions, including oxidative defense, aging, inflammation, redox signaling, cell cycle, and carcinogenesis [27]. This finding revealed that Park7, Pebp1, and Prdx1 in collected lymph were possible diagnostic markers of injured individuals.

TIH is caused by a complex interaction of numerous processes, involving vascular endothelial injury, platelet hyperactivity, excessive release of procoagulants, hyperfibrinogenemia, anticoagulant pathways impairment, and fibrinolysis shutdown [4]. Immunoregulatory platelet dysfunction is the main pathological mechanism of TIH, despite the platelet count is at a normal level [1, 28]. On the one hand, injury-induced platelet activation promotes platelets to bind with leukocytes, forms platelet-leukocyte aggregates and activates innate immune response [29, 30]. On the other hand, activated neutrophils and macrophages release extracellular traps to simulate platelet aggregation and thrombin formation [1]. Therefore, we assumed that thrombolysis therapy reduced not just TIH but also excessive immune response. Figure 2C shows that the lymph of the CLO group had much lower levels of pro-inflammatory and immune-associated proteins, as confirmed by the top 5 GO terms of BP. Except for Q5WRG2 (Angiogenin, Ang) and Q6P6T1 (Complement C1s subcomponent, C1s) were previously reported to be presented in both plasma and lymph after trauma [14], we discovered following new protein molecules of lymph, including P50115 (Protein S100-A8, S100a8), P52925 (High mobility group protein B2, Hmgb2), F7FP65 (Retinoic acid receptor responder protein 2, Rarres2), D3ZWD6 (Complement C8 alpha chain, C8a), P01836 (Ig kappa chain C region, A allele), P00697 (Lysozyme C-1, Lyz1), G3V9C7 (Histone H2B, Hist1h2bk), M0R485 (Peptidoglycan recognition protein 2, Pglyrp2), P55314 (Complement component C8 beta chain, C8b), Q91YB6 (Complement inhibitory factor H, Cfh), D3ZPI8 (Complement C8 gamma chain, C8g). Although these down-regulated proteins are linked to inflammatory and immunological responses, it’s unclear if they’re just found in lymph fluid. Hence, more study is needed to identify particular lymph biomarkers following TIH or TIH plus thrombotic treatment.

### Insight of lymph metabolome

The metabolomic researches of trauma-induced hemorrhagic shock are continuously reported [31–35], while the metabolomic changes of TIH are still little known. We depicted the lymph metabolomics of TIH and the potential pharmaceutical effect of antithrombotic therapy.

Compared to the sham group, homocystine, the up-regulated metabolite in the lymph of the VEH group, is enriched in cysteine and methionine metabolism (Fig. 3B). Homocystine is synthesized via transmethylation of methionine and enzymatic reaction [36]. Homocystine is regarded as an independent risk factor for thrombotic disorders and cardiovascular disease [37–39]. In addition, cysteine and methionine metabolism is also significantly increased in the plasma metabolome of injured animals and patients [31–35]. This result indicates that homocystine of lymph and plasma might be a potential biomarker of TIH.

Lysophosphatidylcholine is the main active component of oxidized low-density lipoprotein and can expand inflammation and exacerbate diseases by inducing the migration of lymphocytes and macrophages to produce pro-inflammatory cytokines [40–43]. Koji et al. detected lysophosphatidylcholine significantly increased in the mesenteric lymph on the model of trauma-induced hemorrhagic shock [43]. To our surprise, compared to the sham group, lysophosphatidylcholine is the down-regulated metabolite in the lymph of the VEH group and enriched in choline metabolism in cancer and glycerophospholipid metabolism (Fig. 3C). This result is opposite to that of previous research probably due to different animal models. Additionally, compared to the VEH group, lysophosphatidylcholine was found to significantly increase in the lymph of the CLO group (Fig. 4B). We inferred that (1) the sham, VEH, and CLO groups induced lysophosphatidylcholine generation due to operation; (2) In VEH group, lysophosphatidylcholine was accumulated in the injured sites and failed to be transported into thoracic duct due to the blockage of lymphatic vessels by lymphatic platelet thrombosis; (3) In CLO group, unblocked lymphatic vessels drained increased lysophosphatidylcholine of injured sites into thoracic duct.

Cholic acid is the main component of bile acids in the human body and has versatile roles in maintaining bile acid homeostasis, alleviating metabolic inflammation, and protecting neural injury [44]. Compared to the VEH group, cholic acid, enriched in bile secretion and primary bile acid biosynthesis, is significantly up-regulated in the CLO group. This result suggests that lymphatic platelet thrombolysis not only alleviates TIH but also improves systematical pathology.

Docosahexaenoic acid is an acknowledged neuroprotective and anti-inflammatory agent with multiple functions of alleviating endoplasmic reticulum and oxidative stress and regulating autophagy [45–48]. N1-Methyl-2-pyridone-5-carboxamide is one of the major metabolites of nicotinamide and is elevated in renal failure, vascular inflammation, chronic ulcerative colitis, and asthma [49–52]. Compared to the VEH group, docosahexaenoic acid, and N1-Methyl-2-pyridone-5-carboxamide are decreased in the CLO group (Fig. 4C). This is probably because antithrombotic therapy improves lymphatic transport of docosahexaenoic acid and N1-Methyl-2-pyridone-5-carboxamide, thus inhibiting their accumulation at fracture sides and decreasing oxidative response and nicotinamide metabolism.

Isoleucine is a kind of branchedchain and an essential amino acid for humans and animals [53]. Isoleucine plays diverse roles in physiological functions and metabolic pathways, including maintaining the growth and development of animals, enhancing immunity, regulating glucose transportation, and stimulating protein synthesis [53–56]. Testosterone is synthesized and secreted by testicular Leydig cells and the adrenal cortex and is regulated by the hypothalamic-pituitary-gonadal axis [57]. Testosterone plays irreplaceable roles in the growth and development of the human body, maintaining musculoskeletal homeostasis and regulating post-traumatic stress disorder [58–60]. Although little literature reports their direct relationships with trauma,  the information above seems to imply that higher levels of isoleucine and testosterone are beneficial for patients with trauma. However, the data of lymph metabolomics indicated that the levels of isoleucine and testosterone in the CLO group were significantly lower than VEH group. Therefore, general analysis suggests that (1) thrombolysis therapy is good for relieving TIH, circulatory system disorder, and inflammatory and oxidative response; (2) but might lead to side effects, such as metabolic and endocrine disorders. In a word, these DEMs above are potential and sensitive biomarkers of TIH patients with antithrombotic therapy.

### Limitations and outlook

TIH is a dynamic, sequential pathophysiological process. Our work focused on a single time point of TIH to evaluate the constitutive alterations of lymph using integrated proteome and metabolome. (2) To distinguish the different and unique biomarkers between blood-derived and lymph-derived samples, a time course co-analysis of plasma and lymph muti-omics in trauma patients is mandatory. (3) Because obtaining lymph is challenging, the previous collection of lymph in trauma patients is under the help of anesthesiologists and intensive care physicians. Multidisciplinary collaboration in clinical trials of trauma patients is required to investigate the relationship between the possible biomarkers and the prognosis of TIH in order to validate the sensitivity and specificity of screening lymph biomarkers in this study. (4) Given that men have a higher risk of traumatic fractures and TIH [61–64], we only employed young male rats in this experiment. To increase the study’s relevance and effect, both genders are suggested to be incorporated into the study design.

### Electronic supplementary material

Below is the link to the electronic supplementary material.

Supplementary Material 1

Supplementary Material 2. Supplemental Fig. 1 Scheme of the methodology. 18 male Sprague Dawley rats were randomly assigned to three groups, respectively sham group (6 non-fractured rats with sham surgery and vehicle-treated ), vehicle group (6 fractured rats with vehicle-treated), and clopidogrel group (6 fractured rats with clopidogrel-treated). Thoracic duct lymph on 24 h post-surgery was collected and centrifuged, the supernatant of lymph was detected by integrated proteomics and metabolomics to comprehensively describe the lymph profile of TIH. DEPs: differentially expressed proteins, DEMs: differentially expressed metabolites.

Supplementary Material 3. Supplemental Fig. 2 Overview of proteomic data of lymph. (A) The number of proteins corresponding to different molecular weight distributions. (B) PCA of identified proteins in Sham, VEH, and CLO groups. (C) Hierarchical clustering dendrogram of sample Euclidean distance in Sham, VEH, and CLO groups. (D) The number of up-regulated and down-regulated DEPs in different groups. (E) Venn plot of the DEPs of lymph in different groups.

Supplementary Material 4. Supplemental Fig. 3 Overview of metabolomic data of lymph. (A) Metabolites intensity distribution of QC samples. (B) The proportion of the identified metabolites in each chemical classification. (C) PCA plot of all samples of lymph. (D) OPLS-DA analysis of lymph. (E) Permutation analysis of lymph. To validate OPLS-DA mode, a cross-validation plot was analyzed by UPLC-Q-TOF/MS-based metabonomic data with 7-fold cross-validation and 200 times response permutation testing. (F) The number of up-regulated and down-regulated DEMs in different groups. (G) Venn plot of the DEMs of lymph in different groups.

Supplementary Material 5. Supplementary Table 1 Differentially expressed proteins identified in rat lymph.

Supplementary Material 6. Supplementary Table 2 Differentially expressed metabolites identified in rat lymph.

## Data Availability

1. The mass spectrometry proteomics data have been deposited to the ProteomeXchange Consortium (http://proteomecentral.proteomexchange.org) via the iProX partner repository with the dataset identifier PXD047692.2. The metabolomic data have been deposited to the MetaboLights (https://www.ebi.ac.uk/metabolights/) with the dataset identifier MTBLS9145.
