# Supplementary figures and images for: Integrated proteomic and metabolomic profiling of lymph after trauma-induced hypercoagulopathy and antithrombotic therapy
Source: Thromb J. 2024 Jul 10;22:59. doi: 10.1186/s12959-024-00634-3 (PMC11234664; doi:10.1186/s12959-024-00634-3)

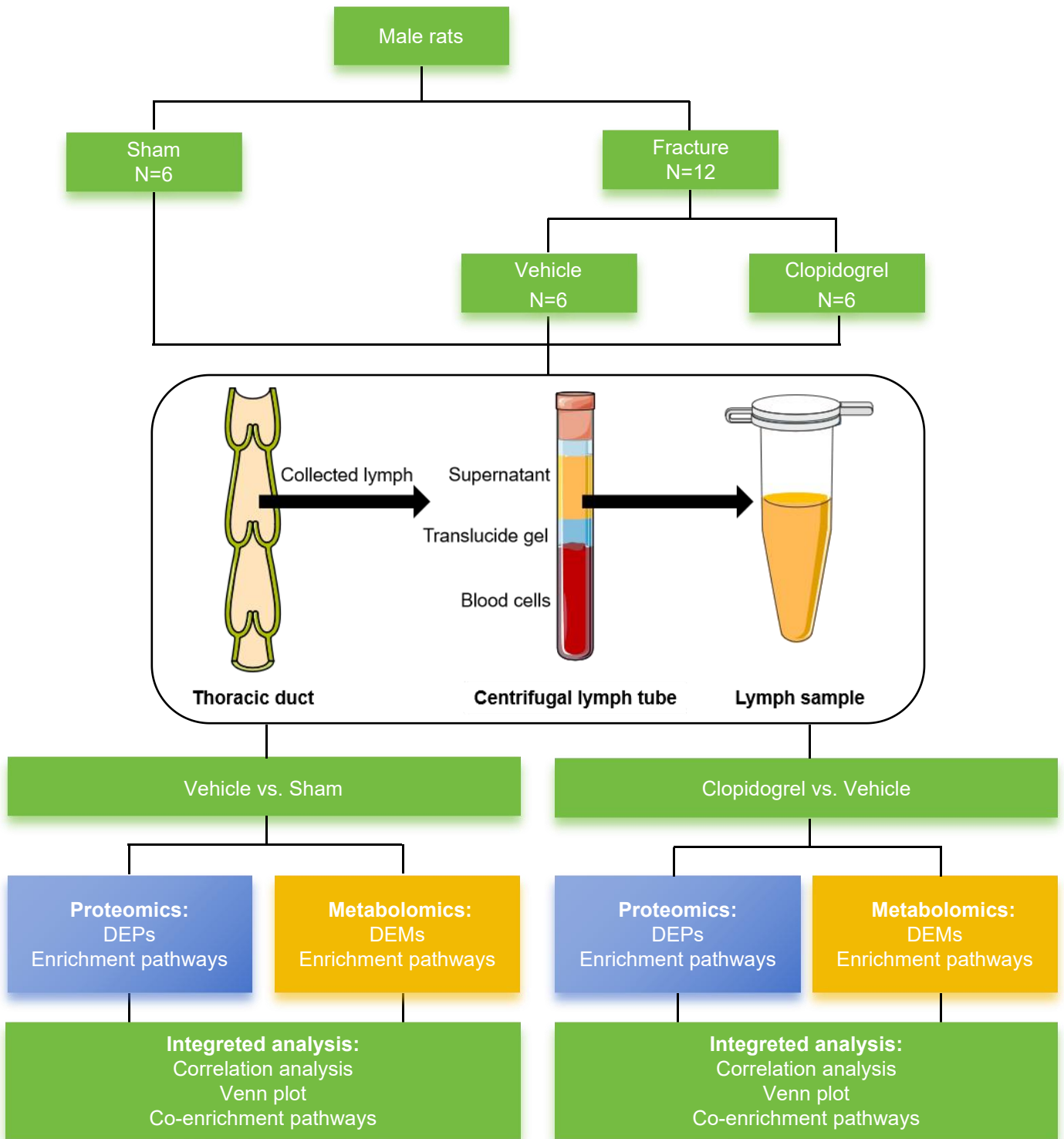

Supplement: Supplementary file 1 — Supplementary Material 1 [file 12959_2024_634_MOESM1_ESM.pdf]

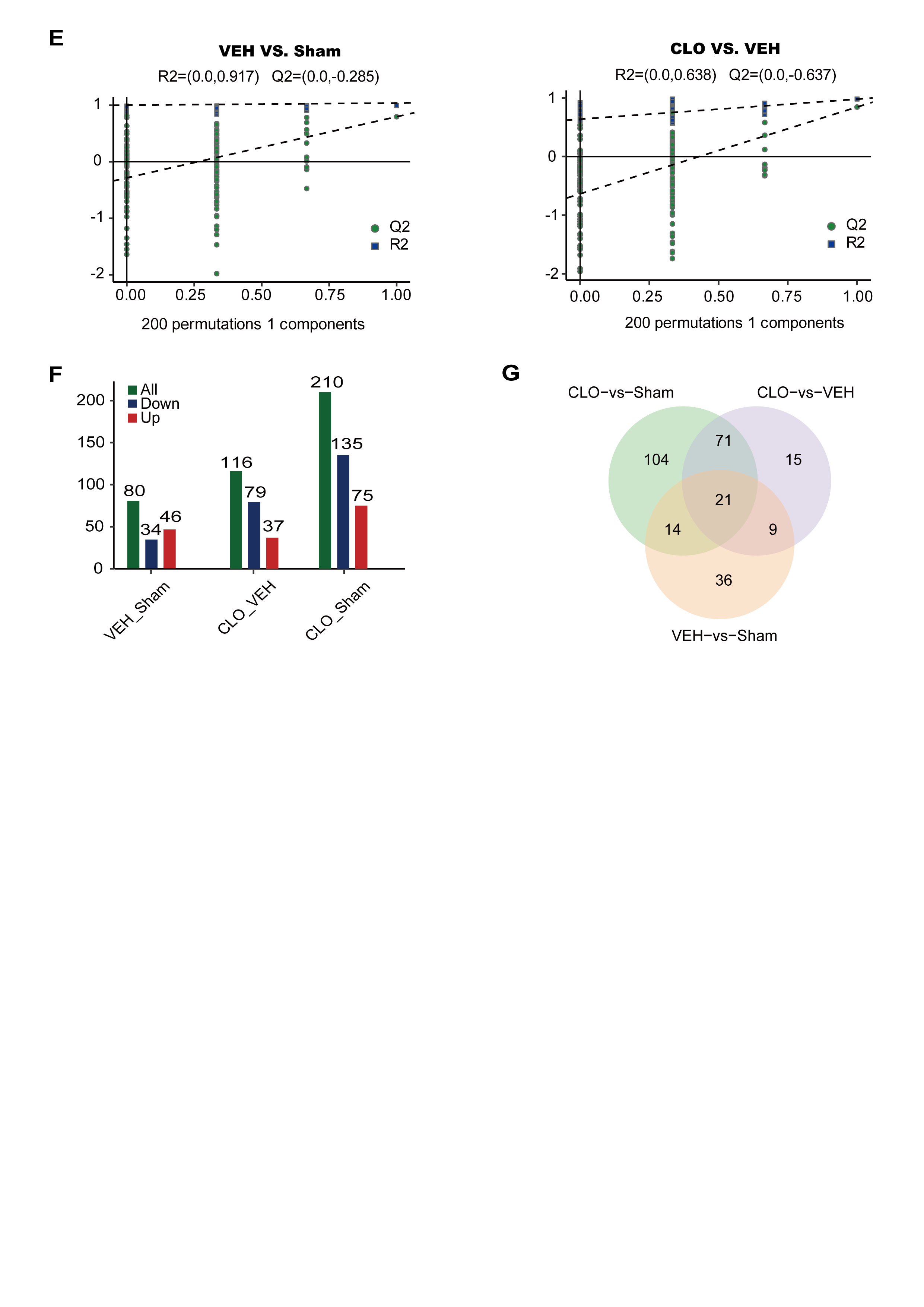

Supplement: Supplementary file 2 — Supplementary Material 2. Supplemental Fig. 1 Scheme of the methodology. 18 male Sprague Dawley rats were randomly assigned to three groups, respectively sham group (6 non-fractured rats with sham surgery and vehicle-treated ), vehicle group (6 fractured rats with vehicle-treated), and clopidogrel group (6 fractured rats with clopidogrel-treated). Thoracic duct lymph on 24 h post-surgery was collected and centrifuged, the supernatant of lymph was detected by integrated proteomics and metabolomics to comprehensively describe the lymph profile of TIH. DEPs: differentially expressed proteins, DEMs: differentially expressed metabolites. [file 12959_2024_634_MOESM2_ESM.tif]

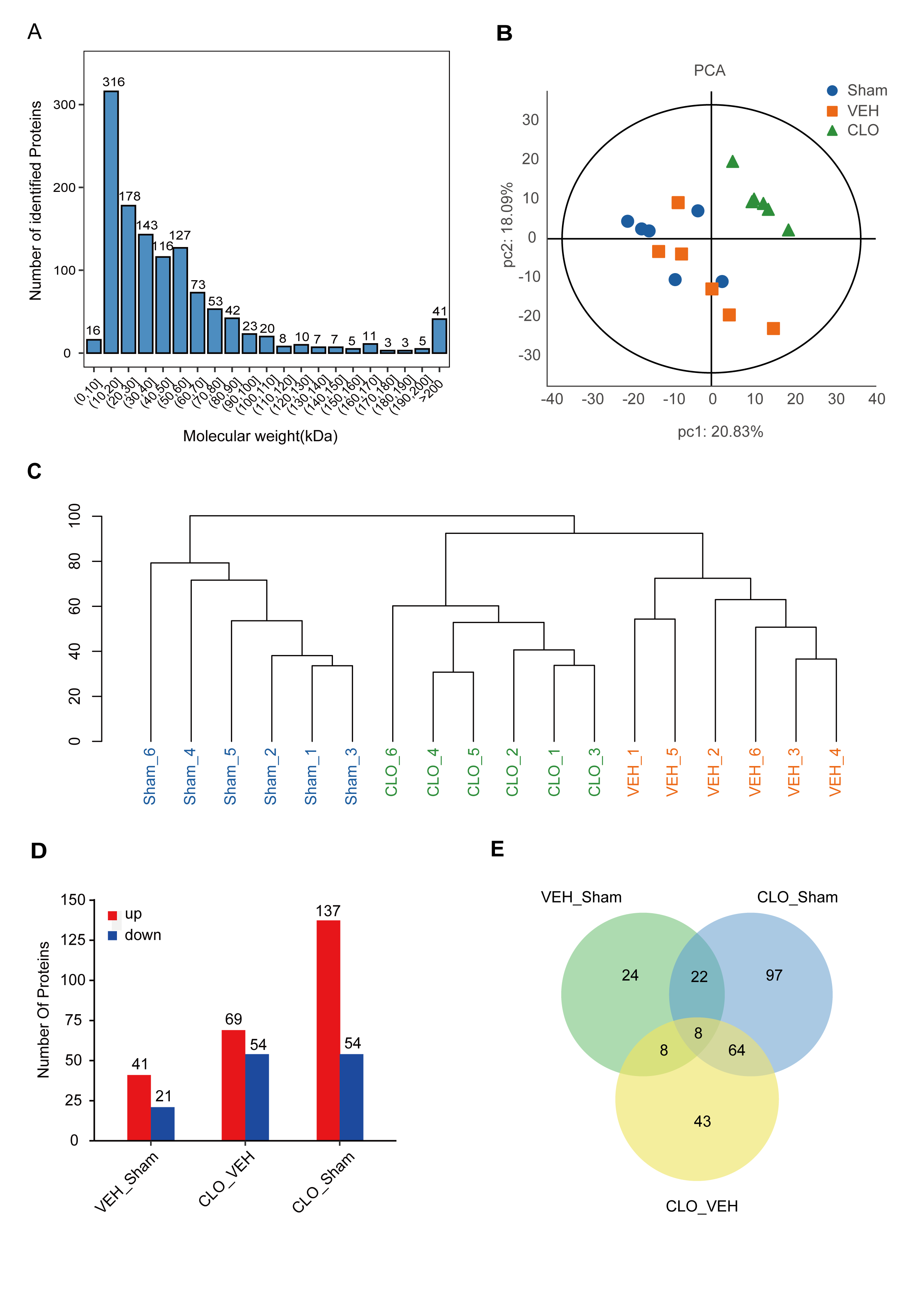

Supplement: Supplementary file 3 — Supplementary Material 3. Supplemental Fig. 2 Overview of proteomic data of lymph. (A) The number of proteins corresponding to different molecular weight distributions. (B) PCA of identified proteins in Sham, VEH, and CLO groups. (C) Hierarchical clustering dendrogram of sample Euclidean distance in Sham, VEH, and CLO groups. (D) The number of up-regulated and down-regulated DEPs in different groups. (E) Venn plot of the DEPs of lymph in different groups. [file 12959_2024_634_MOESM3_ESM.tif]

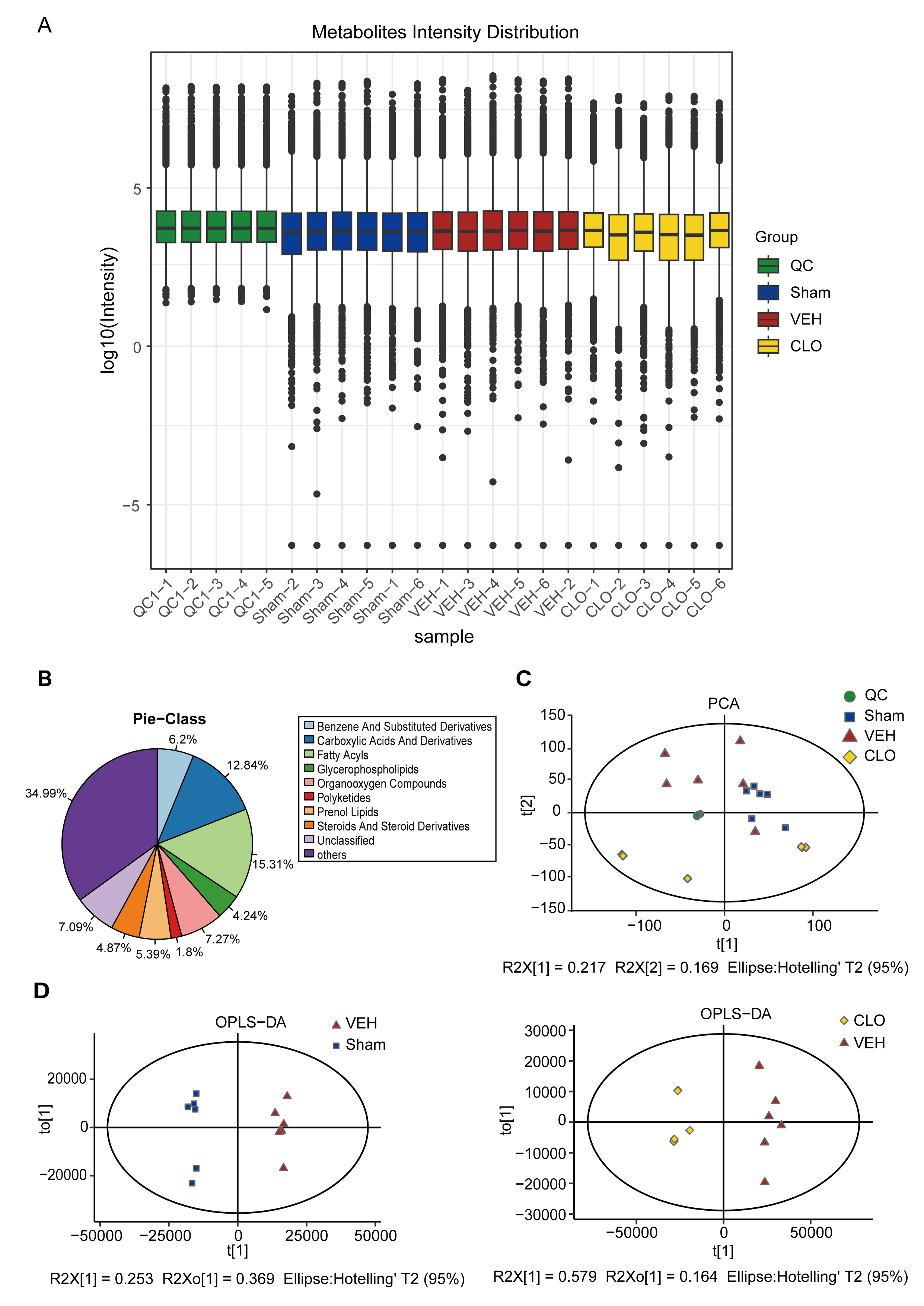

Supplement: Supplementary file 4 — Supplementary Material 4. Supplemental Fig. 3 Overview of metabolomic data of lymph. (A) Metabolites intensity distribution of QC samples. (B) The proportion of the identified metabolites in each chemical classification. (C) PCA plot of all samples of lymph. (D) OPLS-DA analysis of lymph. (E) Permutation analysis of lymph. To validate OPLS-DA mode, a cross-validation plot was analyzed by UPLC-Q-TOF/MS-based metabonomic data with 7-fold cross-validation and 200 times response permutation testing. (F) The number of up-regulated and down-regulated DEMs in different groups. (G) Venn plot of the DEMs of lymph in different groups. [file 12959_2024_634_MOESM4_ESM.tif]
